# Effectiveness of Surgical Sealants in Reducing Prolonged Air Leaks After Pulmonary Resection: A Systematic Review

**DOI:** 10.1093/icvts/ivag164

**Published:** 2026-06-03

**Authors:** Aleena Chinoy, Alan Soo

**Affiliations:** Cardiothoracic Surgery Department, Galway University Hospital, Galway, H91 YR71, Ireland; University of Galway, University Road, Galway, H91 TK33, Ireland; Cardiothoracic Surgery Department, Galway University Hospital, Galway, H91 YR71, Ireland; University of Galway, University Road, Galway, H91 TK33, Ireland

**Keywords:** prolonged air leak, pulmonary resection, surgical sealants, lung surgery

## Abstract

**Objectives:**

Prolonged air leak (PAL) is the most common complication following pulmonary resection, leading to increased morbidity, hospital stay, and healthcare costs. Although staplers are currently the standard for lung parenchymal closure, ALs remain frequent. Surgical sealants have been introduced as adjuncts to staplers to reduce PAL, but evidence remains inconsistent. Prior systematic reviews frequently pooled together stapled and sutured resections, limiting applicability to contemporary practice. This review specifically evaluates whether surgical sealants reduce the incidence and duration of PAL when used with staplers in adult patients.

**Methods:**

This systematic review followed the Preferred Reporting Items for Systematic Reviews and Meta-Analyses (PRISMA) guidelines and was registered on the International Prospective Register of Systematic Reviews (PROSPERO) (CRD420251064592). A literature search was conducted in PubMed, Ovid MEDLINE, and Cochrane Library for studies published between 2005 and June 2025. Eligible studies included adult patients undergoing stapler-based pulmonary resection comparing sealants plus staplers versus staplers alone. Risk of bias was assessed using the revised Cochrane risk-of-bias tool for randomized trials (RoB2) and risk of bias in non-randomized studies of interventions (ROBINS-I) tools.

**Results:**

Eight studies were included. Surgical sealants studied included fibrin-based (autologous fibrin sealant (FS), FS patch, human FS) and synthetic polymers (polyethylene glycol hydrogel sealant, cyanoacrylate-based sealant). Most studies reported a statistically significant reduction in PAL incidence and/or duration with sealant use. Some also showed reduced chest tube duration and hospital stay, though not always statistically significant. One study showed worse outcomes in the sealant group. No study reported increased complications. Subgroup analyses were limited.

**Conclusions:**

Sealants used with staplers may reduce PAL incidence and duration, particularly in high-risk patients, without increased complications. However, variability in study design and limited subgroup data weaken current evidence. Larger, standardized randomized controlled trials are needed to confirm clinical benefit and inform routine use.

## Graphical abstract

**Figure:**
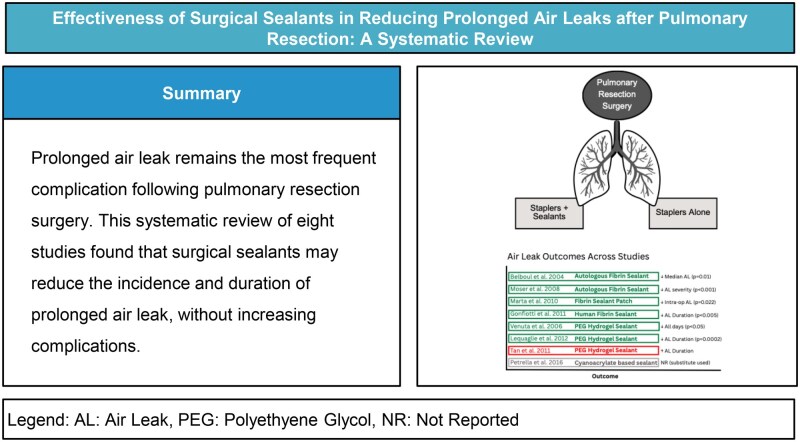

## INTRODUCTION

Prolonged air leak (PAL) remains the most frequent complication following pulmonary resection surgery and represents a significant burden for both patients and healthcare systems. Prolonged air leak is defined as air leakage from the lung parenchyma persisting beyond 5 to 7 days postoperatively and occurs in up to 20% of patients, particularly following lobectomy or segmentectomy.^1^^,^^2^ Although intraoperative ALs are observed in up to 48% to88% of resections, most resolve spontaneously within several days.^3^^,^^4^ Persistent air leaks, however, are associated with increased risk of infection, prolonged hospital stay, higher healthcare costs, and overall increased morbidity.^1^^,^^5–7^

Patients with chronic obstructive pulmonary disease (COPD) or emphysema are at increased risk due to the fragility and decreased elasticity of their lung tissue.^4^^,^^8^ Intraoperative factors such as incomplete interlobar fissures, pleural adhesions, and extensive parenchymal manipulation further contribute to PAL risk.^1^

Numerous strategies have been proposed to mitigate PAL, including pleural tenting in upper lobe resections, fissure-less lobectomy, the use of pneumoperitoneum, and reinforced or buttressed staplers.^1^^,^^2^^,^^9^ Digital AL monitoring systems, such as *DigiVent* and *Thopaz*, allow objective quantification of ALs and may reduce unnecessary chest drainage.^1^^,^^10^ Despite these advances, PAL remains a common postoperative complication, particularly in high-risk patient populations.^4^^,^^5^^,^^6^

Surgical staplers have now become the predominant method for lung parenchymal division, largely replacing hand-sewn techniques due to their reliability, efficiency, and reduced technical variability.^5^^,^^11^ However, ALs persist even with these modern stapling devices. Surgical sealants, both fibrin-based and synthetic polymer-based, have therefore been developed as adjuncts to staplers to reinforce staple lines and enhance parenchymal closure.^1^^,^^4^ Their routine use remains controversial due to cost considerations and inconsistent trial results.^2^^,^^12^

Previous systematic reviews have reported mixed findings and frequently combined stapled and sutured resections, limiting relevance to modern thoracic practice.^12^ Given the widespread adoption of staplers, a focused evaluation of sealants used specifically in conjunction with staplers is warranted to better inform surgical decision-making, particularly in patients at increased risk of PAL.

The objective of this systematic review was to evaluate whether the use of surgical sealants in conjunction with staplers, compared with staplers alone, reduces the incidence and duration of PAL in adult patients undergoing pulmonary resection.

## METHODS

This systematic review was conducted in accordance with the Preferred Reporting Items for Systematic Reviews and Meta-Analyses (PRISMA) guidelines.^13^ The primary outcomes assessed are the incidence and duration of PAL, with secondary outcomes including chest tube duration, length of hospital stay, postoperative complications, and subgroup effects in patients with pulmonary comorbidities. By restricting inclusion to stapler-based resections, this review addresses limitations of previous literature and provides a focused assessment of surgical sealant effectiveness applicable to contemporary thoracic surgical practice.

All included studies involved adult patients who underwent pulmonary resection surgery using staplers. The intervention assessed in this review was the application of surgical sealants in combination with staplers, compared to the use of staplers alone. The primary outcomes of interest were the incidence and duration of PAL. Secondary outcomes included chest drain duration, length of hospital stay, postoperative complications, and any subgroup outcomes based on comorbidities such as COPD or emphysema.

A systematic review was conducted as it allows for a comprehensive and structured synthesis of existing evidence. Given the variability in outcomes, surgical techniques, and definitions of PAL in the current literature, a systematic review provides the most effective method to aggregate data, identify consistent patterns, and highlight gaps to inform future research and clinical decision-making.

This systematic review protocol has been registered in International Prospective Register of Systematic Reviews (PROSPERO) 2025 (CRD420251064592). A computer-based literature search was performed from 2005 to June 2025, in the following databases: Cochrane Library, PubMed, and Ovid MEDLINE. The exact search strategy used is available in the registry and in **Appendix S1**. Medical Subject Headings (MeSH) terms were selected based on terminology used in previously published systematic reviews and primary studies on surgical sealants and pulmonary resection.^12^ The final literature search was completed in June 2025.

### Inclusion and exclusion criteria

#### Inclusion criteria

- Studies published from 2005 and June 2025
- English language studies with full text availability
- Adult patients (>18 years) undergoing pulmonary resection (lobectomy, segmentectomy, or wedge resection) for either malignant or benign pulmonary conditions
- Stapler-based pulmonary resections
- Comparison of surgical sealants plus staplers versus staplers alone
- Reporting at least 1 of the following outcomes:
  - Incidence of PAL
  - Duration of AL
  - Length of hospital stay
  - Postoperative complications
  - Subgroup outcomes based on patient comorbidities.

#### Exclusion criteria

- Full text not available
- Paediatric studies (<18 years)
- Animal or in vitro studies
- Pneumonectomy or redo surgery on the same side
- Use of sutures, buttressing or mesh
- Unclear specification of stapler or suture use
- Studies comparing different sealants without a stapler-only control
- Case reports or studies without clear outcome reporting.

### Risk of bias assessment

Risk of bias was assessed for all papers included in this review. The revised Cochrane risk-of-bias tool for randomized trials (RoB2) was used for all randomized controlled trials (RCTs).^14^ For the non-randomized studies, the risk of bias in non-randomized studies of interventions (ROBINS-I) tool was used.^15^

## RESULTS

**Figure 1.**
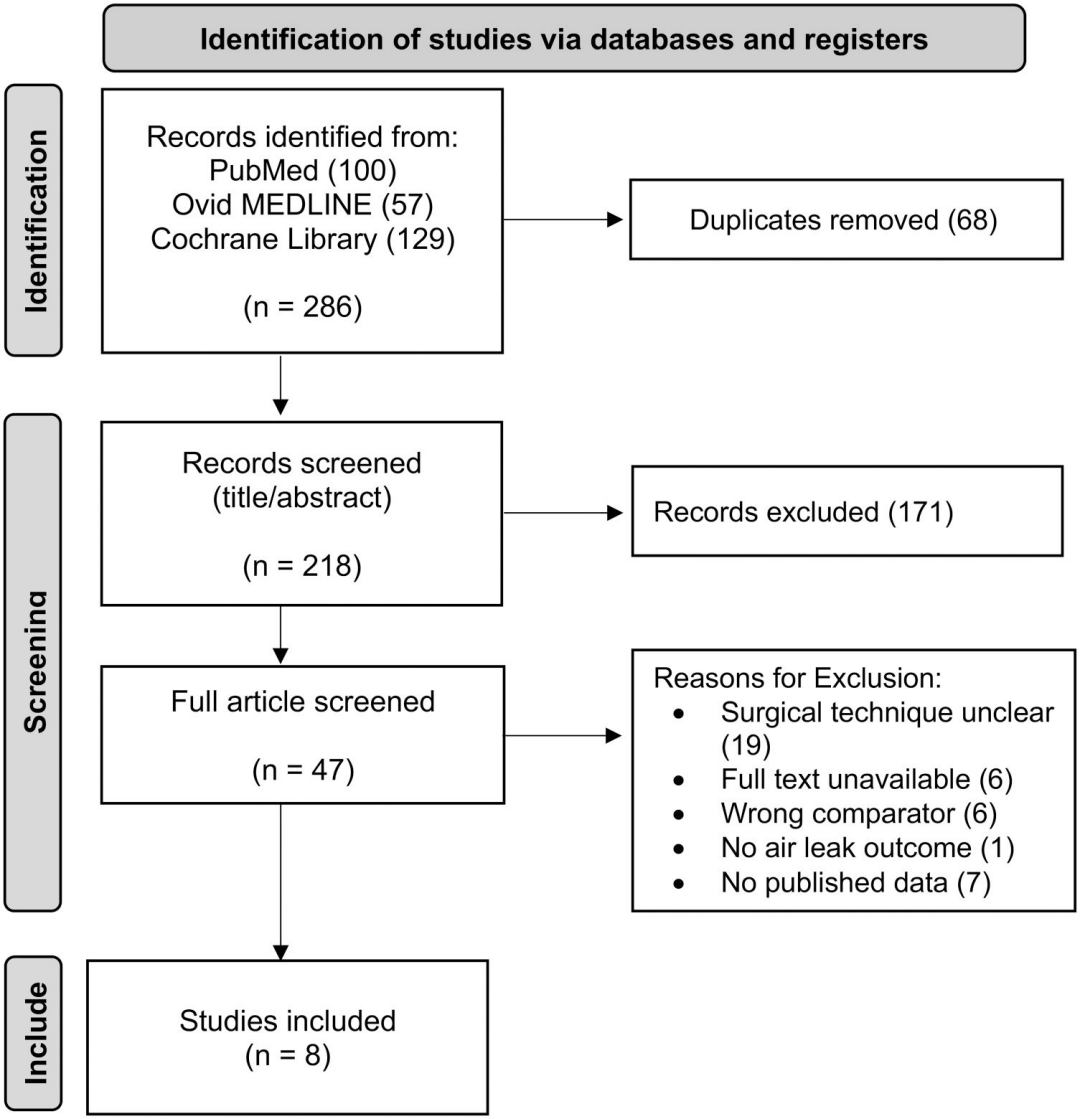
PRISMA Flow Diagram

The electronic database search identified a total of 286 papers. After the removal of duplicates, 218 titles and abstracts were screened, and 47 full-text articles were assessed for eligibility. Rayyan was used for screening, enabling 2 independent reviewers to evaluate the papers for eligibility. Any disagreement was resolved by discussion and consensus. Following exclusion based on the criteria outlined above, 8 papers were included in the final review. One paper was initially published in 2004; however, it was added to CENTRAL in 2005, so it has been included in this review.^16^ The selection process of the included studies is highlighted in **Figure 1**.

Eight studies met the inclusion criteria, comprising 7 RCTs, all of which received ethical approval and informed consent from their participants, and 1 case-control study. The papers included are shown in **Table 1**, and the characteristics of the studies are detailed in **Appendix S2**. The included papers review the effectiveness of multiple surgical sealants, including autologous fibrin sealants (FSs) (Vivostat, Vivostat A/S), FS patches (TachoSil, Nycomed), human FSs (Kedrion SpA), polyethylene glycol (PEG) hydrogel sealants (CoSeal, Baxter Biosurgery Europe), and cyanoacrylate-based sealants (Innoseal, Innoseal Europe B.V.).

**Table 1.** Papers Included in This Systematic Review

No.	Author	Year	Title and journal	Sealant	Study type
1	Belboul et al	2004	“The effect of autologous fibrin sealant (Vivostat) on morbidity after pulmonary lobectomy: a prospective randomized, blinded study” *European Journal of Cardiothoracic Surgery*	Autologous fibrin sealant	RCT
2	Venuta et al	2006	“Use of a polymeric sealant to reduce air leaks after lobectomy” *The Journal of Thoracic and Cardiovascular Surgery*	PEG sealant	RCT
3	Moser et al	2008	“Autologous fibrin sealant reduces the incidence of prolonged air leak and duration of chest tube drainage after lung volume reduction surgery: A prospective randomized blinded study” *The Journal of Thoracic and Cardiovascular Surgery*	Autologous fibrin sealant	RCT
4	Marta et al	2010	“Efficacy and safety of TachoSil versus standard treatment of air leakage after pulmonary lobectomy” *European Journal of Cardiothoracic Surgery*	Fibrin sealant patch	RCT
5	Gonfiotti et al	2011	“Safety and Effectiveness of a New Fibrin Air Leak Sealant: A Multicentre, Controlled, Prospective, Parallel-Group, Randomized Clinical Trial” *The Annals of Thoracic Surgery*	Human fibrin sealant	RCT
6	Tan et al	2011	“A prospective randomized controlled study to assess the effectiveness of Coseal to seal air leaks in lung surgery” *European Journal of Cardiothoracic Surgery*	PEG sealant	RCT
7	Lequaglie et al	2012	“Use of a sealant to prevent prolonged air leaks after lung resection: a prospective randomized study” *Journal of Cardiothoracic Surgery*	PEG sealant	RCT
8	Petrella et al	2016	“Efficacy and Safety of Innoseal for air leak after pulmonary resection: a case control study” *Journal of Surgical Research*	Cyanoacrylate-based sealant	Case control

Abbreviations: PEG = polyethylene glycol; RCT = randomized controlled trial.

Risk of bias was assessed for all the papers. Six RCTs were found to have some concerns,^1–3^^,^^9^^,^^10^^,^^16^ 1 RCT was low risk,^4^ and the case control study^17^ was found to have a moderate risk of bias (see **Appendix S3**).

All included studies investigated the use of surgical sealants applied in conjunction with staplers during pulmonary resections, comparing outcomes to staplers alone. The surgical sealants assessed include fibrin-based sealants or synthetic polymer-based adhesives. Sample sizes ranged from 25 to 299 patients. Three of the included studies involved patients undergoing lobectomy only, while 3 studies included mixed pulmonary resections, including lobectomy, bi-lobectomy, and sublobar resections. One study focused exclusively on patients undergoing lung volume reduction surgery (LVRS). A summary of all the included studies is outlined in **Table 2**. Seven studies reported measures of AL incidence and duration, while 1 study did not directly report these outcomes but assessed AL-related outcomes using chest tube duration as a surrogate measure. Chest tube duration and length of hospital stay were reported in 7 studies.

**Table 2.** Summary of Included Studies Evaluating Surgical Sealants in Pulmonary Resection

Study	Sealant	Design/population	Intervention/control (n)	PAL incidence/air leak outcome	Air leak duration	Chest tube duration	Length of hospital stay	Key notes
Belboul et al. 2004	Autologous FS	RCT; lobectomy	20/20	Reduced postoperative air leak (40% vs 80%; *P* = .02)	Reduced (*P* = .01)	Shorter, not significant	Shorter, not significant	Significantly reduced drainage volume (*P* < .001)
Venuta et al. 2006	PEG sealant	RCT; lobectomy	25/25	Reduced PAL incidence (8% vs 20%; *P* < .05)	Reduced	Reduced (*P* = .03)	Reduced (*P* = .009)	-
Moser et al. 2008	Autologous FS	RCT (intra-patient); LVRS	25 (intra-patient)	Reduced PAL incidence (4.5% vs 18.2%; *P* = .031)	Reduced (*P* < .001)	Reduced (*P* < .001)	Not reported	Severe emphysema cohort; 12% mortality
Marta et al. 2010	FS patch	RCT; lobectomy	148/151	Reduced postoperative air leak (*P* = .022; *P* < .03)	Reduced (*P* = .014)	No significant difference	No significant difference	-
Gonfiotti et al. 2011	Human FS	RCT; pulmonary resections	91/94	Reduced postoperative air leak (*P* < .001)	Reduced (*P* < .005)	No significant difference	No significant difference	–
Tan et al. 2011	PEG sealant	RCT; pulmonary resections	61/60	No reduction in PAL	No significant difference (*P* = .09)	Increased (4 vs 3 days)	Increased (7 vs 6 days)	Only study reporting worse outcomes with sealant
Lequaglie et al. 2012	PEG sealant	RCT; pulmonary resections	111/111	Reduced PAL incidence (*P* = .0013)	Reduced (*P* = .0002)	Not reported	Reduced (*P* < .0001)	-
Petrella et al. 2016	Cyanoacrylate-based sealant	Case control; pulmonary resections	30/30	Not reported (air leak grade used for inclusion)	Not reported	Reduced (*P* = .005)	No significant difference (*P* = .0762)	FEV1 subgroup analysis (not significant)

Abbreviations: FS, fibrin sealant; LVRS, lung volume reduction surgery; NA = not applicable; PAL, prolonged air leak; PEG, polyethylene glycol; RCT, randomized controlled trial.

### Incidence and duration of PAL

All included studies assessed the AL duration following pulmonary resection. Seven of the 8 studies reported on a statistically significant reduction in AL duration or surrogate measures in patients receiving surgical sealants in addition to staplers, compared to staplers alone, while 1 study reported no significant reduction.

Belboul et al^16^ reported a significantly lower rate of immediate postoperative AL in the sealant group (*P* = .02), alongside a reduced median AL duration (*P* = .01). Venuta et al^9^ found a significantly lower daily rate of ALs in the intervention group across all postoperative days (*P* < .05 for each day), and a reduced incidence of PAL (8% vs 20%). Moser et al,^4^ using an intra-patient design in severe emphysema patients undergoing bilateral LVRS, demonstrated a significantly lower total AL severity score (*P* < .001) and a lower incidence of PAL (*P* = .031) on the treated side. Marta et al^3^ observed a significant reduction in both intraoperative AL (*P* = .022) and postoperative AL (*P* = .03) in the FS patch group. Gonfiotti et al^10^ found more patients without AL at wound closure in the sealant group (*P* < .001), and significantly shorter postoperative AL duration (*P* < .005). Similarly, Lequaglie et al^1^ reported significantly fewer postoperative ALs (*P* = .0002) and a lower incidence of PAL (*P* = .0013) in the PEG sealant group. Although Petrella et al^17^ did not directly report AL rates, they used time to chest tube removal as a substitute, showing a statistically significant benefit in the sealant group. Only Tan et al reported a longer AL duration in the sealant group, contrary to findings in the rest of the studies. However, this was not statistically significant.^2^

### Chest tube duration

Reductions in chest tube duration were reported in 7 of the included studies. Venuta et al^9^ observed a significant reduction (*P* = .03), while Moser et al^4^ also found a shorter drainage duration on the sealant-treated side (*P* < .001). Petrella et al^17^ reported a significantly faster time to chest drain removal in the cyanoacrylate-based sealant group (3.5 vs 5 days, *P* = .005). Although Marta et al^3^ noted a reduction in chest drain duration (median 4 vs 5 days), the difference was not statistically significant (*P* = .054). Belboul et al^16^ reported significantly lower drain output volume (*P* < .001), though chest tube duration did not differ. Gonfiotti et al^10^ and Tan et al^2^ both reported no benefit in terms of chest tube duration, with Tan noting a longer duration in the sealant group (4 vs 3 days).

### Length of hospital stay

Hospital length of stay was variably reported. Lequaglie et al^1^ documented a statistically significant reduction in hospital stay in the PEG sealant group (4 vs 8 days, *P* < .001). Venuta et al^9^ also found a shorter hospital stay in the sealant group (*P* = .009). Petrella et al^17^ observed a nonsignificant shorter stay in the sealant group (5 vs 6 days, *P* = .0762). Marta et al^3^ also reported on a reduction that was not statistically significant (*P* = .35). Belboul et al,^16^ Gonfiotti et al,^10^ and Tan et al^2^ found no statistically significant differences, with Tan et al once again reporting a slightly longer stay in the sealant group (7 vs 6 days). Moser et al did not report on hospital stay duration.^4^

### Postoperative complications

None of the included studies reported an increased rate of complications in the sealant group. All sealants were well tolerated with no serious adverse events attributed to their use.

### Subgroup analyses

Only Petrella et al^17^ performed subgroup analysis based on preoperative pulmonary function (forced expiratory volume in 1 second [FEV1]) but found no significant differences in outcomes between groups. While most other studies reported baseline equivalence in patient characteristics between intervention and control arms, none stratified results by comorbidities such as COPD or emphysema, despite these being established risk factors for prolonged AL. However, the study by Moser et al^4^ specifically included patients with severe emphysema undergoing LVRS, thereby providing evidence on sealant effectiveness within this high-risk subgroup.

## DISCUSSION

This systematic review synthesized current evidence on the use of surgical sealants as adjuncts to staplers in reducing PAL after pulmonary resection. Overall, the findings support the use of sealants, whether fibrin-based or synthetic polymers, as potentially beneficial in reducing the incidence and duration of PAL. Importantly, none of the included studies reported a significant increase in postoperative complications, supporting the safety of these adjuncts.

Previous systematic reviews and meta-analyses evaluating the use of surgical sealants for the prevention of prolonged AL have reported mixed results. Many of these reviews included heterogeneous surgical techniques, frequently combining stapled and sutured resections, which limits their applicability to contemporary thoracic practice. In contrast, the present review focuses exclusively on stapler-based pulmonary resections, allowing for a more targeted assessment of sealant effectiveness within modern surgical workflows. This methodological distinction may account for differences between the findings of this review and those reported in earlier analyses.

Prolonged air leak is one of the most common complications following pulmonary resection surgery and is associated with increased morbidity, longer hospital stays, and greater healthcare costs. However, a key challenge in interpreting the literature is the lack of a standardized definition for PAL. Some studies define PAL as an AL lasting more than 5 days, while others use a 7-day threshold. This inconsistency complicates direct comparison and meta-analysis, highlighting the need for consensus definitions in future trials. In addition, the included studies encompassed different types of pulmonary resections, including lobectomy, mixed pulmonary resections, and 1 study focusing on lung volume resection surgery. This procedural heterogeneity is clinically relevant, as the incidence and severity of PAL are known to vary between resection types, particularly in patients with emphysema or reduced pulmonary reserve.^8^ As a result, direct comparison across studies and the generalizability of uniform conclusions regarding PAL prevention should be interpreted with caution.

Multiple different surgical sealants were assessed in the included studies. The autologous fibrin-based sealant, Vivostat, is produced from 120 mL of the patient’s own blood intraoperatively.^4^^,^^16^ It was initially used for haemostasis in cardiac surgery; however, it has also demonstrated a statistically significant reduction in PAL in both Moser and Belboul studies. Moser et al^4^ also evaluated the autologous FS specifically in patients with severe emphysema undergoing LVRS, providing insight into high-risk populations. Marta et al^3^ assessed a collagen patch coated in human thrombin and fibrinogen that reported significant reductions in both intraoperative and postoperative ALs, with no increase in adverse events. The FS patch has been approved by the European Medicines Agency (EMA) for haemostasis and tissue sealant in vascular surgery and to prevent cerebrospinal fluid (CSF) leakage in neurosurgery.^18^ The Kedrion SpA FS is a newer sealant consisting of human fibrinogen, thrombin, and bovine aprotinin. The authors confirmed its viral inactivation process and safety and reported a higher proportion of patients without AL at wound closure and shorter AL duration.^10^ The use of a PEG sealant was assessed in 3 studies. This synthetic PEG-based hydrogel binds to tissue through cross-linking proteins and is fully resorbed within 30 days.^1^^,^^2^ It has been approved by the Food and Drug Administration (FDA), indicated in vascular surgery for haemostasis and to seal leakages.^19^ Both Lequaglie et al^1^ and Venuta et al^9^ demonstrated significant reductions in PAL with this PEG sealant. However, Tan et al reported contrary findings, with longer AL duration and hospital length of stay in the PEG sealant group. Notably, this study included patients with mild (grade 1) ALs, which typically resolve spontaneously and may have influenced these outcomes. In Tan et al,^2^ ALs were graded intraoperatively during lung reinflation to 25 cmH_2_O, with grade 1 defined as a single stream of bubbles, grade 2 as 2 to 5 streams, and grade 3 as confluent bubbling. The last sealant assessed in the studies was cyanoacrylate-based sealant, composed of n-butyl-2-cyanoacrylate, n-octyl-2-cyanoacrylate, and vitamin E which forms a fast-acting adhesive on contact with tissue.^17^ Petrella et al’s^17^ case-control study showed shorter chest tube duration and earlier discharge in the cyanoacrylate-based sealant group, even though the study design was non-randomized.

Most of the included RCTs were small in scale and carried some concerns regarding risk of bias, particularly related to the measurement of outcome and blinding. Only Moser et al^4^ was assessed as low risk. Seven out of the 8 included studies reported significant reductions in PAL incidence or duration. Importantly, none reported increased postoperative complications, reinforcing the overall safety of sealant use.

Despite these positive findings, the limited number of high-quality studies restricts the ability to draw definitive conclusions. Only 2 studies performed subgroup analysis; Petrella et al^17^ stratified outcomes by FEV1, while Moser et al^4^ exclusively enrolled patients with severe emphysema. Given that comorbidities such as COPD and emphysema are well-established risk factors for PAL, future trials should prioritize stratified analysis based on pulmonary function or disease severity.

The findings of this systematic review align broadly with prior literature. A 2010 Cochrane review published in 2010 included 16 RCTs and found that while surgical sealants reduced the incidence of PAL in most trials, there was only a reduction in length of hospital stay in 3 trials. They concluded that the routine use of surgical sealants could not yet be recommended for all patients, though there may be an indication for sealants in some cases.^12^ This review pooled data from studies using both sutures and staplers. However, as modern thoracic surgery has shifted almost exclusively to stapler-based resections, this systematic review focuses exclusively on the adjunctive use of sealants with staplers alone. This focused approach yields insights that are more directly applicable to modern surgical practice.

While reductions in PAL incidence and duration were consistently reported, findings for chest tube duration and length of hospital stay were more variable. Some studies reported significant reductions, while others did not. These differences may be due to variations in discharge protocols, chest tube removal criteria, and institutional practices, making standardized outcome reporting essential in future trials.

Although several included studies demonstrated reductions in prolonged AL incidence and chest tube duration with the use of surgical sealants, these improvements did not consistently translate into shorter hospital stays or reductions in postoperative complications. Length of stay varied across studies, and reported postoperative morbidity was generally low, suggesting that factors beyond AL alone, such as institutional discharge protocols, perioperative pathways, and patient comorbidity, may influence these outcomes. Consequently, while reductions in AL-related measures may offer procedural or logistical benefits, their overall clinical impact should be interpreted with caution.

A 2006 European Association for Cardio-Thoracic Surgery- European Society of Thoracic Surgeons (EACTS-ESTS) survey showed that 49% of surgeons believed sealants were clinically beneficial, while 34% were uncertain and 17% believed they were ineffective. Only 8% used them routinely, due to cost and availability barriers.^20^ Given the expanding literature on the topic and the introduction of various newer and more effective sealants, an updated survey would be valuable to assess how surgical attitudes have evolved since 2006.

The most recent study included in this review was published in 2016, reflecting the limited availability of contemporary randomised data evaluating surgical sealants in pulmonary resection. While a comprehensive search was performed, no more recent studies meeting the inclusion criteria were identified. Advances in surgical technique and perioperative care over the past decade, including the widespread adoption of video-assisted and robotic-assisted thoracic surgery, may influence the current applicability of these findings. As such, the results of this review should be interpreted in the context of evolving surgical practice.

### Limitations

Several limitations were observed across the included studies. Sample sizes were small in most trials, limiting statistical power. There was an inconsistency in the definitions of PAL, limiting direct comparison of the studies. The studies raised some concerns regarding risk of bias, particularly related to the measurement of outcomes and blinding. Subgroup analyses were limited, restricting insight into the effectiveness of surgical sealants in high-risk patient cohorts, such as those with COPD or emphysema. Although patients with severe emphysema are at a particularly high risk of PAL, only 1 included study specifically evaluated this population. Consequently, the limited number of emphysema-focused studies restricts the ability to draw subgroup-specific conclusions regarding sealant effectiveness in this setting.

### Future recommendations

Several ongoing trials are currently investigating newer sealants and their application. It is essential for trials to focus on the application of sealants in high-risk populations, as they have the potential for the greatest benefit to this adjunctive treatment. Ideally, future research should consist of larger, multicentre, adequately powered RCTs with standardized definitions of PAL, consistent outcome reporting. Subgroup analyses should be pre-specified and stratified by comorbidities, including emphysema and COPD, to better guide targeted clinical application. Future studies should also incorporate cost-effectiveness analyses, as the high cost of surgical sealants remains a key limitation to their widespread clinical use. In addition, further studies focusing on high-risk emphysema cohorts would be valuable to better define the role of sealants in this population.

## CONCLUSION

This systematic review found that the use of surgical sealants in conjunction with staplers during pulmonary resection is generally associated with a reduction in the incidence and duration of prolonged ALs. Most included studies demonstrated beneficial effects without increased postoperative complications. However, variability in study design, definitions of PAL, and limited subgroup analysis reduces the strength of the current evidence.

Sealants appear to be a safe and potentially effective adjunct, particularly in patients at higher risk of PAL. Nonetheless, further RCTs with standardized outcome reporting and stratified analyses are needed to confirm these findings and refine patient selection.

## AUTHOR CONTRIBUTIONS

Aleena Chinoy was responsible for all aspects of the study, including conceptualization, data extraction, analysis, interpretation, and writing of this paper. Professor Alan Soo was responsible for the conceptualization, supervision, review, and editing of this paper.

## Supplementary Material

ivag164_Supplementary_Data

## Data Availability

The data underlying this article are available in the article and in its online supplementary material.

## Abbreviations

AL: air leak
COPD: chronic obstructive pulmonary disease
CSF: cerebrospinal fluid
EACTS: European Association for Cardio-Thoracic Surgery
EMA: European Medicines Agency
ESTS: European Society of Thoracic Surgeons
FDA: Food and Drug Administration
FEV1: forced expiratory volume in 1 second
FS: fibrin sealant
LVRS: lung volume reduction surgery
MeSH: Medical Subject Headings
NR: not reported
NS: not significant
PAL: prolonged air leak
PEG: polyethylene glycol
PRISMA: Preferred Reporting Items for Systematic Reviews and Meta-Analyses
PROSPERO: International Prospective Register of Systematic Reviews
RCT: randomized controlled trial
RoB2: revised Cochrane risk-of-bias tool for randomized trials
ROBINS I: risk of bias in non-randomized studies of interventions
