# Supplementary material for: Effectiveness of Surgical Sealants in Reducing Prolonged Air Leaks After Pulmonary Resection: A Systematic Review
Source: Interdiscip Cardiovasc Thorac Surg. 2026 Jun 3;41(7):ivag164. doi: 10.1093/icvts/ivag164 (PMC13353221; doi:10.1093/icvts/ivag164)

## **Appendix**

**Appendix 1: Full search strategy**

**PubMed:** ("Thoracic Surgery"[MeSh] OR "Pulmonary Surgical Procedures"[MeSh] OR lobectomy[tiab] OR segmenectomy[tiab] OR "wedge resection"[tiab] OR "lung resection"[tiab] OR "pulmonary resection"[tiab]) AND ("Surgical Staplers"[MeSH] OR stapler*[tiab] OR "Tissue Adhesives"[MeSH] OR sealant*[tiab] OR "surgical glue"[tiab] OR "fibrin glue"[tiab] OR "fibrin sealant"[tiab] OR "biological adhesive"[tiab] OR "tissue glue"[tiab] OR TachoSil[tiab] OR BioGlue[tiab] OR Coseal[tiab]) AND (air[tiab] AND (leak*[tiab] OR leakage[tiab])

**Cochrane Library:** (pulmonary resection OR lung resection OR lobectomy OR segmentectomy OR wedge resection) AND (stapler OR surgical staplers OR sealant OR surgical glue OR fibrin glue OR fibrin sealant OR biological adhesive OR tissue adhesive OR TachoSil OR BioGlue OR Coseal) AND (air leak OR prolonged air leak OR air leakage OR postoperative complication) in Title Abstract Keyword - (Word variations have been searched)

**OVID – MEDLINE**

1 Thoracic Surgery/ 14101

2 pulmonary surgical procedures.mp. or Pulmonary Surgical Procedures/ 4355

3 lobectomy.mp. 24490

4 wedge resection.mp. 5078

5 lung resection.mp. 5998

6 pulmonary resection.mp. 4842

7 segmentectomy.mp. 4972

8 1 or 2 or 3 or 4 or 5 or 6 or 7 55311

9 air leak.mp. 3273

10 Surgical Staplers/ or staplers.mp. 5117

11 Fibrin Tissue Adhesive/ or sealants.mp. or Tissue Adhesives/ 16370

12 10 or 11 21418

1. 8 and 9 and 12 93
2. limit 13 to (english language and humans and yr="2005 -Current") 57

**Appendix 2: Characteristics of included studies (ordered by year of publication)**

**Belboul 2004**

| Methods | A prospective randomised, blinded study |
| --- | --- |
| Participants | 40 patients undergoing elective pulmonary lobectomy were included and randomised into two groups. 20 patients were assigned to autologous fibrin sealant group and 20 patients were assigned to the control group. |
| Interventions | 1. Intervention group: Autologous fibrin sealant (Vivostat) 2. Control group: Staplers only, no additional intervention |
| Outcomes | - Air leak on the day of surgery and daily until chest drain was removed - Time to chest drain removal - Chest drain output - Duration of thoracic epidural analgesia treatment - Postoperative length of stay |
| Notes | All patients had lung tumours limited to one lobe with no established nodal disease. Air leak was assessed intraoperatively and all patients had air leaks before randomisation. Sealant prepared from 120ml of the patient’s own blood and applied intraoperatively. |
| Conclusion | Vivostat significantly reduced air leaks and drainage volume. |

**Venuta 2006**

| Methods | A prospective randomised, controlled study |
| --- | --- |
| Participants | 50 patients undergoing pulmonary lobectomy for lung cancer were included and randomised into two groups. 25 patients were assigned to surgical sealant group and 25 patients were assigned to the control group. |
| Interventions | 1. Intervention group: Surgical sealant (Coseal) 2. Control group: Staplers only, no sealant used |
| Outcomes | - Air leak presence and duration - Chest tube drainage time - Postoperative length of stay |
| Notes | All patients had incomplete fissures. GIA 75 or EndoGia 45 staplers were used. |
| Conclusion | Coseal reduced air leak duration and hospital stay. |

**Moser 2008**

| Methods | A prospective, randomised, intra-patient comparison trial |
| --- | --- |
| Participants | Twenty five patients with severe emphysema undergoing bilateral lung volume reduction surgery (LVRS). |
| Interventions | 1. Intervention group: Autologous fibrin sealant (Vivostat) applied to one lung 2. Control group: No sealant applied to contralateral lung |
| Outcomes | - Postoperative air leak duration and intensity (per side) - Chest tube drainage - Length of hospital stay - Adverse events |
| Notes | LVRS performed bilaterally by video-assisted thoracoscopy and 60mm and 45mm stapler devices. Each person was both treated and the control. |
| Conclusion | Vivostat reduced air leak duration and hospital stay. |

**Marta 2010**

| Methods | A prospective, randomised, open-label, parallel group multicenter trial |
| --- | --- |
| Participants | 299 patients undergoing elective pulmonary lobectomy were included. 148 randomised into TachoSil group and 151 randomised to control group. |
| Interventions | 1. Intervention group: TachoSil 2. Control group: Standard treatment (additional stapling, suturing or no further treatment) |
| Outcomes | - Duration of air leak - Reduction in intraoperative air leak intensity - Time to chest drain removal - Postoperative complications - Length of hospital stay - Pneumothorax/incomplete lung inflation |
| Notes | Air leak was assessed intraoperatively following primary stapling and limited suturing and those with air leak grade 1 or 2 were included and randomised. |
| Conclusion | TachoSil reduced intraoperative air leak intensity and postoperative air leak duration. |

**Gonfiotti 2011**

| Methods | A prospective, randomised controlled trial |
| --- | --- |
| Participants | 185 patients undergoing lobectomy were included. 91 participants randomised to fibrin sealant group and 94 to the control group. |
| Interventions | 1. Intervention group: Fibrin sealant 2. Control group: Standard treatment (no mesh or sealant) |
| Outcomes | - Duration of air leak - Chest tube duration - Hospital length of stay - Postoperative complications |
| Notes | Air leak was assessed intraoperatively and those with grade 1 to 3 were included. |
| Conclusion | Fibrin sealant reduced air leak duration and is safe and effective. |

**Tan 2011**

| Methods | A prospective, randomised controlled trial |
| --- | --- |
| Participants | 200 enrolled, 121 patients included due to presence of air leak. 61 in sealant group, 60 in control group |
| Interventions | 1. Intervention group: Coseal 2. Control group: Standard treatment, no sealant |
| Outcomes | - Duration of air leak - Chest tube duration - Hospital length of stay - Complications |
| Notes | Air leak was assessed intraoperatively following primary stapling and limited suturing and those with air leak grade 1 to 3 were included and randomised. |
| Conclusion | Coseal group had a longer duration of air leak so it is not recommended. |

**Lequaglie 2012**

| Methods | A prospective, randomised controlled trial |
| --- | --- |
| Participants | 1080 participants undergoing elective lung resection (lobectomy, bilobectomy, segmentectomy, decortication) were enrolled. 222 patients were randomised, 111 to CoSeal and 111 to the control group. 105 included in control group after dropouts. |
| Interventions | 1. Intervention group: CoSeal applied to staple lines 2. Control group: Standard treatment |
| Outcomes | - Incidence of PAL (>5 days) - Length of hospital stay - Postoperative complications and costs |
| Notes | Air leak was assessed intraoperatively following primary stapling and those with moderate to severe air leak were randomised. Incidence of postoperative AL was measure with DigiVent. |
| Conclusion | Coseal reduced both overall and PAL and shortened hospital stay. |

**Petrella 2016**

| Methods | A prospective, case-control study with retrospective matched cohorts |
| --- | --- |
| Participants | 30 patients undergoing lobectomy or segmentectomy were enrolled. |
| Interventions | Innoseal application |
| Outcomes | - Duration of air leak - Chest tube duration - Postoperative complications - Length of hospital stay |
| Notes | Those with grade 1 to 2 intraoperative air leak were included and matched 1:1 according to gender, surgical procedure, age and preoperative FEV1. |
| Conclusion | Innoseal effective in treating limited intraoperative air leaks and prevents prolonged postoperative air leaks. |

**Appendix 3: Risk of Bias Assessment**

RoB 2: Risk of bias tool for randomised controlled trials ^(16)^


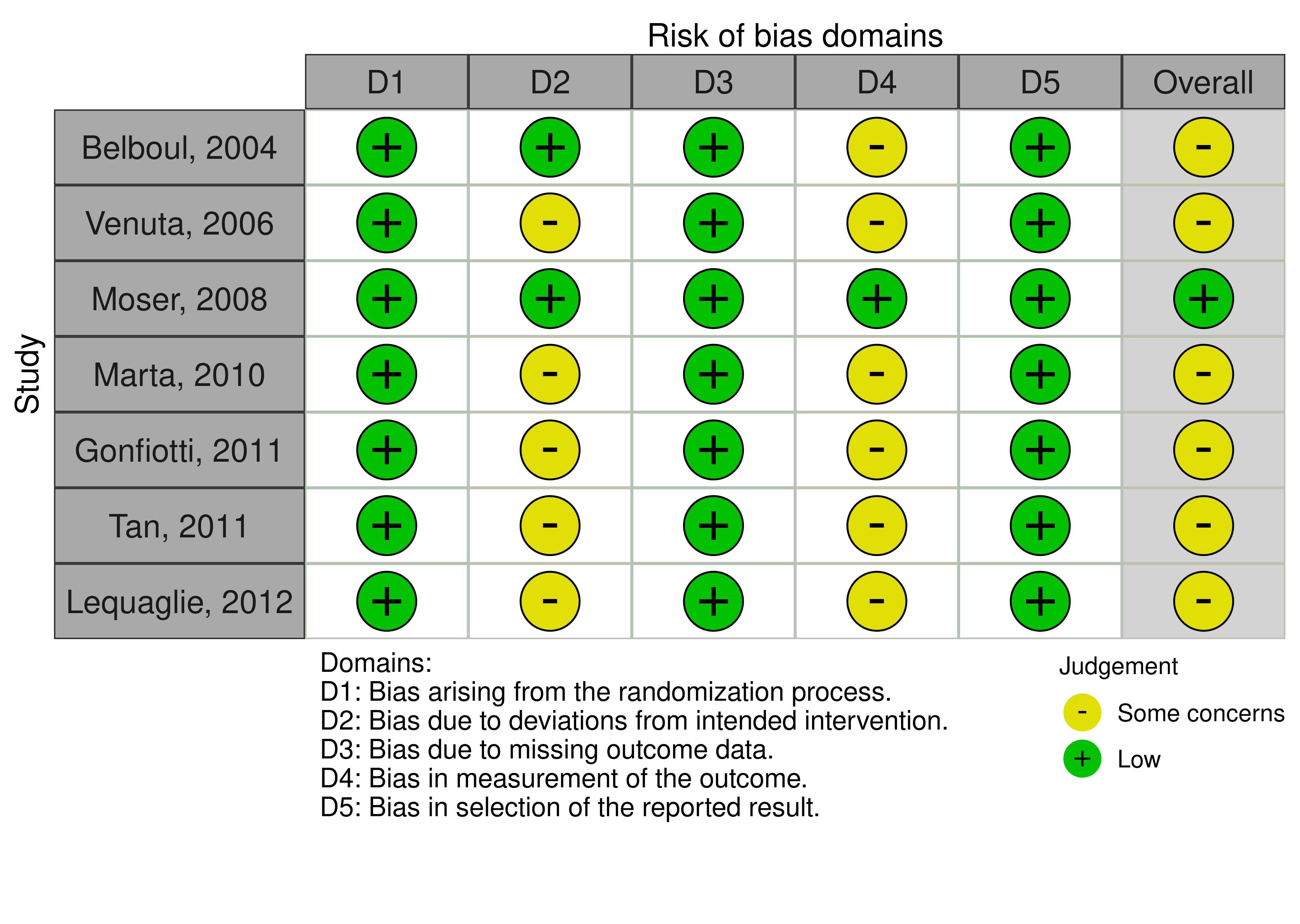


ROBINS-I: Risk of bias tool for non-randomised studies ^(16)^


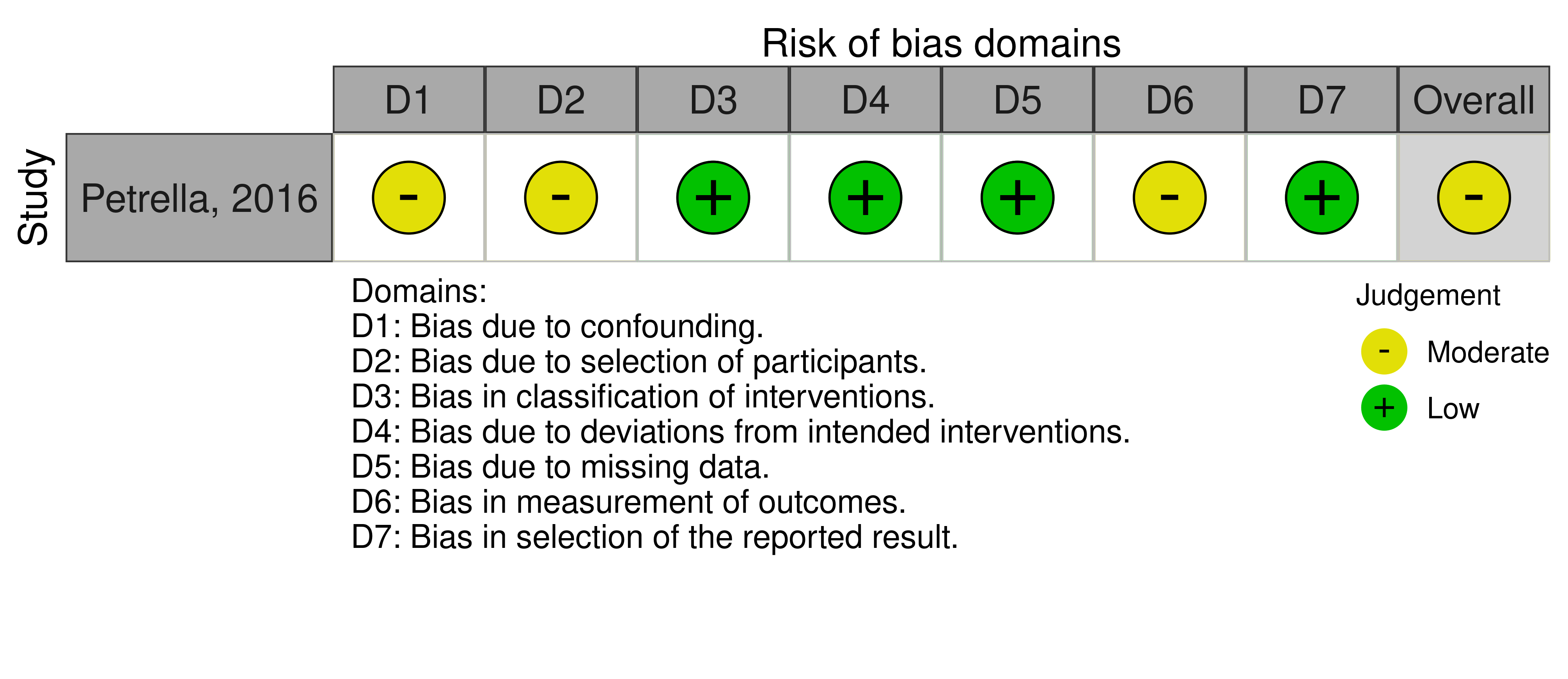

Supplement: ivag164_Supplementary_Data [file ivag164_supplementary_data.docx]
